# Genome-Wide Analysis of *Drosophila* RBf2 Protein Highlights the Diversity of RB Family Targets and Possible Role in Regulation of Ribosome Biosynthesis

**DOI:** 10.1534/g3.115.019166

**Published:** 2015-05-20

**Authors:** Yiliang Wei, Shamba S. Mondal, Rima Mouawad, Bartek Wilczyński, R. William Henry, David N. Arnosti

**Affiliations:** * Department of Biochemistry and Molecular Biology, Michigan State University, East Lansing, Michigan 48824-1319; ‡ Cell and Molecular Biology Program, Michigan State University, East Lansing, Michigan 48824-1319; † Laboratory of Molecular Neurobiology and Laboratory of Bioinformatics, Nencki Institute of Experimental Biology, Pasteura 3, 02-093 Warsaw, Poland,; § Institute of Informatics, University of Warsaw, Banacha 2, 02-097 Warsaw, Poland

**Keywords:** retinoblastoma, RBf1, RBf2, *Drosophila*, ribosomal protein gene

## Abstract

RBf2 is a recently evolved retinoblastoma family member in *Drosophila* that differs from RBf1, especially in the C-terminus. To investigate whether the unique features of RBf2 contribute to diverse roles in gene regulation, we performed chromatin immunoprecipitation sequencing for both RBf2 and RBf1 in embryos. A previous model for RB−E2F interactions suggested that RBf1 binds dE2F1 or dE2F2, whereas RBf2 is restricted to binding to dE2F2; however, we found that RBf2 targets approximately twice as many genes as RBf1. Highly enriched among the RBf2 targets were ribosomal protein genes. We tested the functional significance of this finding by assessing RBf activity on ribosomal protein promoters and the endogenous genes. RBf1 and RBf2 significantly repressed expression of some ribosomal protein genes, although not all bound genes showed transcriptional effects. Interestingly, many ribosomal protein genes are similarly targeted in human cells, indicating that these interactions may be relevant for control of ribosome biosynthesis and growth. We carried out bioinformatic analysis to investigate the basis for differential targeting by these two proteins and found that RBf2-specific promoters have distinct sequence motifs, suggesting unique targeting mechanisms. Association of RBf2 with these promoters appears to be independent of dE2F2/dDP, although promoters bound by both RBf1 and RBf2 require dE2F2/dDP. The presence of unique RBf2 targets suggest that evolutionary appearance of this corepressor represents the acquisition of potentially novel roles in gene regulation for the RB family.

Retinoblastoma (RB) tumor suppressor proteins, including vertebrate RB, p130, and p107, are important regulators of the cell cycle, apoptosis, differentiation, genomic stability, and metabolism (Weinberg 1995; Dyson 1998; Norton *et al.* 1998; Fan and Steer 1999; Zheng and Lee 2002; Hernando *et al.* 2004; Giacinti and Giordano 2006; Nicolay *et al.* 2013; Reynolds *et al.* 2014; and references therein). These proteins function as transcriptional corepressors that bind to E2F and DP proteins and control transcription of a diverse set of target genes, in many cases in a cell cycle−dependent manner (reviewed in Weinberg 1995; Classon and Harlow 2002; Du and Pogoriler 2006; Van den Heuvel and Dyson 2008; and references therein). The *Drosophila* retinoblastoma family members RBf1 and RBf2 are structurally similar to the vertebrate proteins and possess functionally conserved activities in control of cell cycle and developmental genes (reviewed in Du and Pogoriler 2006). The RB-E2F pathway is conserved in most eukaryotic lineages, especially in multicellular organisms (Cao *et al.* 2010). Most arthropod genomes encode a single *RB* gene, which is easily distinguishable by conserved sequences encoding the core “pocket domain” essential for E2F interaction. Interestingly, the genus *Drosophila* contains an additional retinoblastoma family member, *RBf2* (Stevaux *et al.* 2002). The RBf2 protein possesses a conserved pocket domain, similar to that of RBf1. It also contains a distinct C-terminus that lacks the conserved instability element, which has been shown to control both stability and activity of RBf1 (Acharya *et al.* 2010; Raj *et al.* 2012). Both RBf1 and RBf2 mediate transcriptional repression; however, these proteins have different inherent ability to interact with E2F proteins; RBf1 has been found to functionally interact with both the activator dE2F1 as well as the repressor dE2F2, whereas RBf2 is found to interact with dE2F2 but not dE2F1 (Frolov *et al.* 2001; Stevaux *et al.* 2002). Cell-based *in vitro* assays suggested RBf1 acts as a strong repressor of dE2F1 targets. By contrast, the action of RBf2 appears to be weaker and requires coexpression of dE2F2 for maximal repression (Stevaux *et al.* 2002).

RBf1 and RBf2 are coexpressed at many points in development, but there are important differences. In contrast to the relatively stable expression of RBf1 during embryonic development, the RBf2 protein levels vary considerably, with a peak at early stages (Stevaux *et al.* 2002; Keller *et al.* 2005). In contrast to broadly overlapping patterns early in embryogenesis, the proteins show tissue-specific expression in the developing central nervous system. The RBf1 and RBf2 proteins are coexpressed in larval imaginal discs, but RBf1 is the main family member expressed in adults with the exception of the ovary, where RBf2 is also expressed at high levels (Stevaux *et al.* 2002; Keller *et al.* 2005). Consistent with its expression profile, RBf2 was found to repress differentiation markers in embryos and ovaries. Although unlike *RBf1* mutants, *RBf2* null flies are viable, *RBf2* mutant females laid eggs at a fourfold greater rate than wild-type individuals (Stevaux *et al.* 2005). Interestingly, this phenotype was not seen in *de2f2* mutant flies (Stevaux *et al.* 2005), although dE2F2 has been suggested to be the mediator of RBf2 interactions with DNA (Stevaux *et al.* 2002).

The genome binding profile of RBf1 has been characterized in both *Drosophila* embryos and larvae, and both studies revealed that RBf1 interacts with numerous genes related to cellular signaling pathways, in addition to previously characterized cell cycle genes (Acharya *et al.* 2012; Korenjak *et al.* 2012). Although the genome-wide binding of RBf2 has not been reported previously, chromatin immunoprecipitation-quantitative polymerase chain reaction (ChIP-qPCR) of individual target genes has revealed that RBf2 is present at RBf1-bound loci, suggesting that these proteins may regularly co-occupy promoter regions (Korenjak *et al.* 2012). Considering the evolutionary conservation within the genus *Drosophila* of *RBf2* and the pervasive co-occupancy of RBf1 and RBf2, the modest phenotype of *RBf2* mutants presents a conundrum regarding the selection pressure for this gene over large evolutionary periods within *Drosophila*.

Previous studies suggested that RBf1 and RBf2 targeting is mediated via dE2F/dDP (Stevaux *et al.* 2002). Biochemical as well as genetic information supports this view; the larval lethality phenotype of *RBf1* inactivation can be rescued by a mutation in dE2F1 that disrupts this protein’s activation domain (Du 2000). In the larva, a *dDP*-null mutation abolishes the genome-wide association of RBf1, as well as the association of RBf2 to several tested target genes (Korenjak *et al.* 2012). In contrast, the mammalian RB protein does not interact exclusively with E2F family proteins but also physically and functionally interacts with diverse transcription factors and regulatory proteins (as reviewed in Classon and Dyson 2001; Morris and Dyson 2001; Chinnam and Goodrich 2011), as well as components of the RNA polymerase I and III basal transcription machinery (Cavanaugh *et al.* 1995; Larminie *et al.* 1997; White 1997; Hirsch *et al.* 2000, 2004; Gjidoda and Henry 2013). RB proteins in flies, worms, and vertebrates frequently are complexed with additional promoter-associated regulatory factors, including components of the evolutionarily conserved (dREAM) complex, which has been shown to regulate developmental gene expression (Korenjak *et al.* 2004; Lewis *et al.* 2004) . In *Drosophila*, a majority of the RBf1-bound regions also are occupied by one or more proteins of this multiprotein complex (Acharya *et al.* 2012). Genetically, the dREAM complex functions not only as a repressor but also appears to recruit insulator proteins to block enhancer activity on divergently transcribed genes (Bohla *et al.* 2014; Korenjak *et al.* 2014).

In mammals, individual RB family proteins have distinct molecular targets. This targeting is influenced by structural differences in the RB proteins, particularly in the C-terminus, which allow them to bind preferentially to distinct E2F factors (Rubin *et al.* 2005; Julian *et al.* 2008; Cecchini and Dick 2011; Dick and Rubin 2013). In *Drosophila*, the C-terminus of RBf2 is structurally divergent from that of RBf1, which affects the regulation of this protein, and potentially influences promoter targeting (N. Raj and R.W. Henry, unpublished data). To determine how this structurally divergent protein interacts with genomic targets, we carried out parallel chromatin immunoprecipitation sequencing (ChIP-seq) analysis of RBf1 and RBf2 in developing embryos, followed by bioinformatic and functional analysis of target genes. Here, we discuss how distinct genome-wide interactions of RBf2 point to possible diversification in functions for these RBf proteins. Ribosomal protein genes are one class not previously considered as RB targets, pointing to a potentially important role in growth control as well as cell cycle regulation. Analysis of newly identified RBf targets suggest that the canonical RB-E2F model may not describe the full spectrum of interactions found for the derived RBf2 protein.

## Materials and Methods

### Chromatin immunoprecipitation followed by lambda exonulease (ChIP-exo)

ChIP-exo was conducted using 12- to 18-hr-old *yw Drosophila melanogaster* embryos (strain *yw^67^*) collected and aged at room temperature. Fixing and chromatin preparation was conducted as described previously (Acharya *et al.* 2012). Immunoprecipitations and sequencing were carried out by Peconic LLC (State College, PA), using highly specific polyclonal rabbit anti-RBf1 (226.5) or anti-RBf2 (4.7) serum raised against C-terminus of RBf1 or RBf2 as described (Keller *et al.* 2005).

### Read mapping, peaking finding, visualization, and annotation

We obtained 13,453,984 reads for RBf1 ChIP-exo and 12,596,328 reads for RBf2 ChIP-exo. Read mapping was conducted by Peconic LLC. using *Drosophila melanogaster* genome version R5/dm3. To identify RBf1 and RBf2 bound regions, assign these peaks to nearest genes, and classify these peaks to specific genomic regions, Hypergeometric Optimization of Motif EnRichment (HOMER) v3.12 software (http://homer.salk.edu/homer/) was used, with default settings for peak calling and annotation (Heinz *et al.* 2010). The peaks were visualized using IGV browser v2.2.5. We identified 2356 peaks for RBf1 ChIP-exo, which were mapped to 1955 genes, and 4708 peaks for RBf2 ChIP-exo, which were mapped to 3945 genes. The RBf1 and RBf2 peaks are shown in Supporting Information, File S1, and associated annotations in File S2. To compare with dE2F1 and dE2F2 targets, peak information from dE2F1 and dE2F2 ChIP-chip in *Drosophila melanogaster* larvae (Korenjak *et al.* 2012) was annotated with HOMER. To compare RBf1 and RBf2 peaks with BEAF-32 peaks, raw bed file data for binding of BEAF-32 protein in 0- to 8–hr-old *Drosophila melanogaster* embryos was obtained from Yang *et al.* (2012) and peaks were calculated by HOMER using default settings. To compare RBf1 and RBf2 targets with human RB targets, human RB and p130 ChIP-seq peaks (Chicas *et al.* 2010) were annotated using HOMER with the hg18 genome, and their association with human ribosomal protein gene promoters was inspected manually by browsing the peak-calling files in IGV browser with hg18 genome. To analyze the association of *Caenorhabditis elegans* RB homolog Lin-35 with ribosomal protein gene promoters, peak-calling file for Lin-35 (Latorre *et al.* 2015) was visualized in IGV browser with WS220 genome, and the ribosomal protein gene promoters were manually inspected for Lin-35 binding. To compare overlapping peaks between different data sets, HOMER was used with overlapping threshold set at 100 bp.

### *De novo* motif searching

To identify motifs associated with RBf1 and RBf2 targets indicated in Figure S4, the sequences of RBf1 and RBf2 binding regions that associate with TSS/promoter (by HOMER default, −1 kb to +100 bp) were extracted from *Drosophila* genome R5/dm3 on the UCSC Genome Browser and subjected to *de novo* motif searching using MEME-ChIP with default settings (Machanick and Bailey 2011).

### Validation of ChIP-exo peaks

To validate the enrichment of RBf1 and RBf2 on their canonical and noncanonical target genes, several genes were selected and association with RBf proteins tested by ChIP-qPCR. Then, 12- to 18-hr *yw Drosophila melanogaster* embryos were used to prepare chromatin for the immunoprecipitation, and three biological replicates were conducted as previously described (Acharya *et al.* 2012). Preimmune sera RBf1-226.0 and RBf2-4.0 were used for negative controls (Keller *et al.* 2005). Oligonucleotides used for qPCR are listed in File S7. To directly compare RBf1, RBf2 targets with dE2F1, dE2F2, and dDP at the same developmental stage, ChIP-qPCR analysis was performed using the RBf1, RBf2 antibodies and preimmune sera as described above, along with dE2F1, dE2F2, and dDP antibodies (gifts from Dr. Nicholas Dyson lab), in 12- to 18-hr embryos.

### Gene ontology (GO) analysis

RBf1 and RBf2 associated genes identified using HOMER were subjected to GO analysis. The enrichment of GO terms was performed using the online tool DAVID (Huang *et al.* 2009a,b) with KEGG_PATHWAY and SP_PIR_KEYWORDS. Eleven annotation clusters were identified for RBf1 and RBf2 targets, three were identified for RBf1-only targets, and 17 were identified for RBf2-only targets. The enrichment scores of the top five annotation clusters for RBf1 and RBf2 targets, and RBf2-only targets were plotted as shown in Figure 2A. The automated gene assignments by HOMER can arbitrarily assign peaks to one of two divergently transcribed genes, although the distance of RBf peak to the more distal TSS may be close enough to be functionally important. Therefore, to identify all genes that may be likely transcriptional targets of the RBf proteins, and to calculate the percentage of genes bound by RBf1 or RBf2 in different functional groups, RBf1 and RBf2 binding regions were manually inspected in the promoter regions of 81 selected cell-cycle genes, 294 signaling pathway genes (Acharya *et al.* 2012), 94 cytoplasmic ribosomal protein (CRP) genes, and 75 mitochondrial ribosomal protein (MRP) genes (Marygold *et al.* 2007). A few additional genes were therefore added to the dataset of RBf1 or RBf2 potential targets from HOMER; the manually inspected results are shown in File S3.

### Reporter constructs and luciferase assay

To analyze RBf1 and RBf2 activity on ribosome protein gene promoters, promoter regions of *RpL37a* −788 to +132, *RpS29* −369 to +60, *mRpS12/tko* −1074 to +155, *mRpL22* −478 to +79, and *mRpL1* −420 to +47 containing RBf1 or RBf2 binding regions with transcription initiation sites were cloned into *Asc*I and *Sal*I sites in pAC2T-luciferase vector (Acharya *et al.* 2010). The *PCNA*-luciferase reporter was used as a positive control (Acharya *et al.* 2010), and promoter region of *RpS14b* −348 to +33 that is bound by neither RBf1 nor RBf2 was used as a negative control. A total of 100 ng of the reporters were cotransfected with 250 ng of pRL-CMV Renilla luciferase reporter and 250 ng pAX-*RBf1* (Acharya *et al.* 2010), or 250 ng pAX-*RBf2*, with or without 200 ng pIE4-*myc-de2f2*. For the control group, equal amounts of pAX were used instead of pAX-*RBf1*, pAX-*RBf2*, or pIE4-*myc-de2f2*. Luciferase assays were conducted as described before with three biological replicates (Acharya *et al.* 2010), a *t*-test was used to analyze the statistical significance. Cloning primers were listed in File S7.

### RNA interference and ChIP

Double-stranded RNA (dsRNA) for *lacZ*, *de2f1*, *de2f2*, *dDP*, and *BEAF-32* were prepared as described previously (Ullah and Buckley 2007) using primers listed in File S7. A total of 40 million *Drosophila* Kc cells were treated with dsRNA at concentration of 10 μg/mL for 4 d. ChIP from Kc cells was performed as described (Hirsch *et al.* 2004). For qPCR analysis shown in Figure 4C, 1 million *Drosophila* S2 cells were treated with dsRNA for *lacZ*, *RBf1*, *RBf2*, *RBf1+RBf2*, and *RBf2+de2f2* at a concentration of 10 μg/mL for 4 d. Total RNA was isolated using TRIzol (Invitrogen), cDNA was prepared using ABI High Capacity cDNA RT Kit (Life Technologies) following the manual with 2 μg of total RNA. Primers for real-time PCR analysis are listed in File S7.

### RNA-seq

The UAS-*RBf1* fly line was constructed as previously described (Zhang *et al.* 2014). *Pendulin*-Gal4 driver line (Stock Number: 113920) and UAS-*GFP* line (Stock Numbers: 35786) were obtained from Bloomington Stock Center. Then, 100−150 wing imaginal discs were dissected from third-instar larvae of *Pen*Gal4 > UAS *RBf1* and *PenGal4* > UAS *GFP* flies. Total RNA was isolated using TRIzol (Invitrogen) followed by cleanup steps using RNeasy Mini kit (QIAGEN). Then, 1−4 µg of total RNA from three biological replicates was collected. Library preparation and sequencing was conducted by the Research Technology Support Facility (Michigan State University) using an Illumina HiSeq2500. All standard libraries were created using Illumina TruSeq kits and reagents following the manufacturer’s protocols. In brief, polyA mRNA was isolated from total RNA, chemically fragmented, and then reverse transcribed to form double-stranded cDNA. The cDNA was then end repaired, A-tailed, adapter ligated, and amplified to create the final library. A bead-based size selection was performed to target final library molecules with a mean size of 500 base pairs. All libraries were then quantified on a Qubit Fluorometer (Life Technologies) and run on an Agilent BioAnalyzer to determine final size and purity of the library. Final concentration was then determined by qPCR using the KAPA Illumina Library Quantification Kit (KAPABiosystems). Libraries were appropriately diluted and loaded onto the flow cell for sequencing on the Illumina HiSeq2500 following the manufacturer’s protocols. RNA-seq reads were mapped using TopHat v2.0.13 and analyzed using Cufflinks v2.2.1 (Trapnell *et al.* 2012). Analyzed results were visualized using R v2.15.3 with CummeRbund package as described (Trapnell *et al.* 2012).

### Data set preparation for STAP analysis

For all (15,829) *D. melanogaster* genes, their locations and DNA sequences from 500 bp upstream to transcription start site (TSS) were retrieved from Flybase and UCSC database (dmel-5.48 Flybase release). For the four functional groups: CRP genes, MRP genes, cell-cycle genes, and signaling pathway genes, the same data were extracted and processed separately. The quantitative ChIP enrichments were calculated from the .wiggle files computed by MACS v1.4.2 (Zhang *et al.*, 2008) by taking a maximum average signal over a sliding window within the 500bp upstream of the TSS both for RBf1 and RBf2 ChIP experiments. Position weight matrices of 127 motifs of transcription factors binding sites (TFBS) compiled from literature were used as motif information and provided in File S8.

### Testing for motif association with ChIP enrichment

The STAP program was used to test which TFBS affinity scores correlate with ChIP enrichment for the DNA sequences upstream of the TSS (He *et al.* 2009). For individual motif analysis, STAP was run with default parameters (sequence file, data file, and motif file) with the option of co-operative binding set to 0 for each of the 127 motifs. The Pearson correlation between predicted binding and observed binding (in the cases of both RBf1 binding and RBf2 binding) for each of the 127 motifs was plotted using Circos (Krzywinski *et al.* 2009).

### Motif strength assessment

Using a pipeline programmed in Python, MAST (Bailey and Gribskov 1998) was run for each of the 127 motifs on the database of 15,829 sequences to obtain each motif’s occurrences, with maximal p-value = 0.0005 and E-value = 10000. All motif occurrences for each TFBS were extracted from the mast output file. Then, we divided the sequences into two groups: co-bound by RBf1 + RBf2 and bound by RBf2 only. This procedure was repeated for the genome-wide set of sequences as well as the ribosome associated sequences only. We compared the distribution of the strength of nonoverlapping binding sites reported by MAST (as p-values). Negative logarithms (-log10) of those p-values (the lower p-value the stronger value, hence the reverse logarithm) were plotted as histograms for both “RBf1+RBf2” and “RBf2-only.” Mann-Whitney U test was performed on the observed two groups with the threshold of one-sided p-value < 0.05.

## Results

### Genome-wide RBf1 and RBf2 association

**Figure 1:**
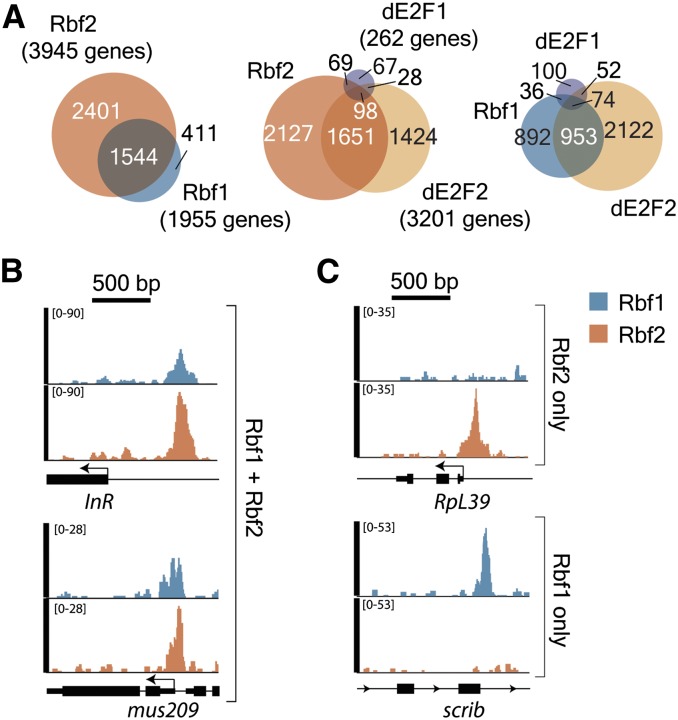
RBf2 binds to a large number of unique targets in the *Drosophila* genome. (A) Visualization of number of genes bound by RBf1 or RBf2 peaks, and overlap of these genes with those targeted by dE2F1 and dE2F2 (Korenjak *et al.* 2012). (B) Examples of promoter regions co-occupied by both RBf1 and RBf2. (C) Examples of genes bound uniquely by RBf1 or RBf2.

To identify the genomic targets of RB family proteins in *Drosophila*, we used ChIP-exo analysis to measure binding profiles for both RBf1 and RBf2 in 12- to 18-hr embryos (Rhee and Pugh 2011; Figure 1A). The canonical RBf−E2F interaction model holds that RBf1 binds to both dE2F1 and dE2F2 proteins whereas RBf2 binds only dE2F2 (Frolov *et al.* 2001; Stevaux *et al.* 2002). Therefore, it was surprising that there were substantially more peaks identified for RBf2 (4708) than for RBf1 (2356); this corresponds to 3945 and 1955 genes, respectively. As noted previously for RBf1, RBf2 binding also was localized primarily to promoter-specific regions (Acharya *et al.* 2012; Figure S1 and Figure S2).

To measure the overlap between the RBf and E2F genomic binding profiles, we compared the RBf genomic targets to those associated with dE2F1 and dE2F2 previously identified in larvae (Korenjak *et al.* 2012). In the larvae, dE2F2 was found to have nearly 4000 binding sites, compared with dE2F1, which only has less than 300 binding sites (Korenjak *et al.* 2012). We mapped the dE2F1 and dE2F2 peaks to the nearest genes and compared with genes bound by RBf1 and RBf2. More than half of RBf1 target genes were bound by dE2F1 or dE2F2, whereas less than half of RBf2 target genes were bound by any E2F factor (Figure 1A). The discrepancy between RBf protein binding and E2F factor binding may reflect the two different developmental stages used for measuring binding, although many individual genes are similarly bound in both stages (Acharya *et al.* 2012; Korenjak *et al.* 2012). To directly compare RBf and E2F targets at the same developmental stage, we conducted ChIP-qPCR analysis using RBf1, RBf2, dE2F1, dE2F2, and dDP antibodies in 12- to 18-hr embryos. We checked selected targets that were previously found bound or not bound by RBf1, RBf2 and dE2F2 (Korenjak *et al.* 2012, and this study) (Figure S3). We noticed weak dE2F2 and dDP bindings on some ribosomal protein gene promoters that were previously shown to be bound by RBf2, but not dE2F2 (Korenjak *et al.* 2012). However, these ChIP signals also were close to signals from nonspecific promoters that were unlikely to be targeted by RBf or E2F (Figure S3). Thus whether these RBf2-only targets are bound by dE2F2 or dDP needs to be determined by the global background of the dE2F2 and dDP antibodies. But it is possible that some RBf2 binding is directed by E2F-independent mechanisms, which we explore below.

RBf1 and RBf2 were found to co-occupy many genes, either through simultaneous binding to multiple transcription factors on a given promoter, or perhaps in a competitive manner (Figure 1B). A small number of promoters were bound only by RBf1 (Figure 1C), whereas others featured significant RBf2 binding and no trace of RBf1, suggesting that these promoters may recruit RBf factors in a different fashion from the genes bound by both RBf1 and RBf2 (Figure 1C). Indeed, motif searches of RBf1/RBf2 peak areas compared to RBf2-only peaks showed that E2F-like sequences were enriched in those areas co-bound by RBf1/RBf2. Motifs enriched under RBf2-alone peaks did not contain E2F-like sequences, but instead contained distinct sequences (Figure S4).

### RBf2 alone targets include most ribosomal protein genes

**Figure 2:**
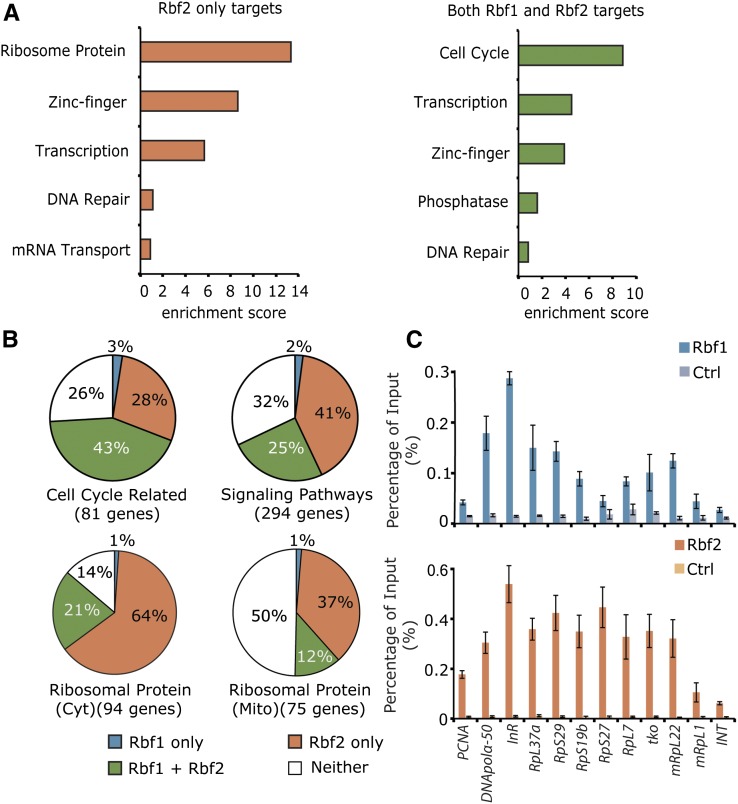
Enriched targeting by RBf1 and RBf2. (A) Genes bound by RBf2 alone or by both RBf1 and RBf2 were functionally annotated using the DAVID database (Huang *et al.* 2009a,b); values indicate enrichment scores. (B) Promoters of cell cycle−related genes (annotated by flybase.org, The Interactive Fly), signaling pathway genes (Acharya *et al.* 2012), cytoplasmic ribosomal protein genes, and mitochondria ribosomal protein genes (Marygold *et al.* 2007) were manually inspected for RBf1 and RBf2 binding sites. Genes indicated in File S3. The signaling pathways include insulin, target of rapamycin (*i.e.*, TOR), PI3K/Akt, 5’ adenosine monophosphate-activated protein , Janus kinase/signal transducers and activators of transcription, transforming growth factor-β, Notch, Wnt, hedgehog, Hippo, nuclear factor-κB, c-Jun N-terminal protein kinases (*i.e.*, JNK), and Ras/Egfr pathways (Acharya *et al.* 2012). (C) To validate ChIP-Seq results, manual chromatin immunoprecipitation of RBf1 and RBf2 on chromatin from 12- to 18-hr embryos was carried out on selected cell-cycle (*PCNA*, *DNApolα-50*), signaling pathway (*InR*), cytoplasmic ribosomal protein (*RpL37a*, *RpS29*, *RpS19b*, *RpS27*, *RpL7*), and mitochondrial ribosomal protein (*mRpS12/tko*, *mRpL22*, *mRpL1*) targets using anti-RBf1, anti-RBf2, and pre-immune serum. An intergenic region (INT) was used as negative control.

We analyzed the nature of genes bound by RBf1, RBf2, or both RBf1/RBf2 using the DAVID gene ontology annotation database (Huang *et al.* 2009a,b). Consistent with the known importance of RB proteins for cell-cycle regulation, genes bound by both RBf1/RBf2 were significantly enriched for this category. In contrast, cell cycle−related genes were not enriched in the set of genes bound solely by RBf2; instead, the most significantly enriched category was that of ribosomal proteins (Figure 2A). The RBf1-only group showed no significant enrichment of any gene class in this analysis (data not shown). To further characterize this enriched feature, we manually inspected RBf1 and RBf2 peaks on each of the 94 CRP gene promoters and 75 MRP gene promoters, observing that RBf2 associated with a majority of the ribosomal protein gene promoters (Figure 2B). We also compared our results with the previous dREAM complex ChIP-chip study (Georlette *et al.* 2007) and found that some, but not all, dREAM complex components co-occupy with RBf1/2 on ribosomal protein gene promoters (Figure S5). In our earlier study (Acharya *et al.* 2012), RBf1 was found to bind multiple genes encoding components of conserved signaling pathways. In the current study, we found that RBf2 also associates with a significant number of signaling pathway gene promoters (Figure 2B). ChIP-qPCR assays were performed on selected cell cycle, signaling pathway, and ribosomal protein targets, confirming the enrichment found in the ChIP-exo experiments (Figure 2C). Thus, RBf2 appears to occupy a greater fraction of noncanonical targets such as signaling pathway and ribosomal protein genes, compared to RBf1, which is present together with RBf2 at many canonical cell cycle−related genes.

### RBf2 shows differential repression activity on ribosomal protein gene promoters

**Figure 3:**
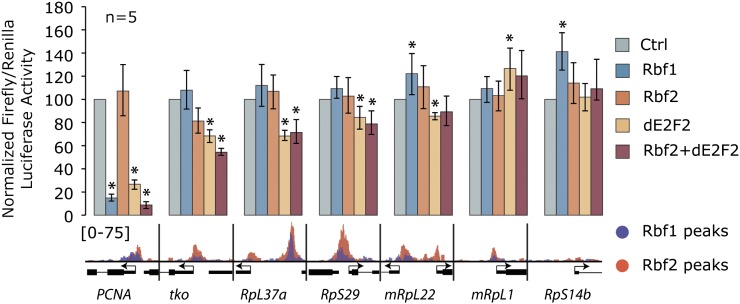
Transcriptional responses of RBf targeted genes in reporter gene assays. RBf1, RBf2, dE2F2, or RBf2/dE2F2 were overexpressed in cells containing reporters with promoter regions of indicated genes. Below, chromatin immunoprecipitation (ChIP) occupancy by RBf1 and RBf2 is shown along with gene structure. RBf1 showed repression activity only on *PCNA*. RBf2 and/or E2F2 significantly repressed *PCNA*, *mRpS12*/*tko*, *RpL37a*, *RpS29*, and *mRpL22*. Activity of *mRpL1* and *RpS14b* promoters was not significantly repressed by any treatment. The increase in expression may be due to indirect effects, particularly for *RpS14b*, which is not bound by these proteins in ChIP assays (*p-value < 0.05). Data represent means and standard deviations from five biological replicates.

To determine the regulatory significance of RBf1 and RBf2 binding at ribosomal protein promoters, we selected several genes for further functional characterization. Six promoter-proximal regions from cytoplasmic and mitochondrial ribosomal protein genes were cloned into a luciferase reporter, and the effects of RBf1, RBf2, dE2F2, or a combination of RBf2 plus dE2F2 were tested in *Drosophila* S2 cells. As expected, transcription from the *PCNA-luc* reporter was repressed by RBf1, dE2F2, and RBf2/dE2F2 (Figure 3). In contrast, none of the ribosomal protein gene promoters were repressed by RBf1, even though these particular promoters have robust RBf1 signals in the embryo. Notably, overexpression of RBf2 alone repressed the *mRpS12/tko* promoter ∼25%, with repression increasing to ∼50% with coexpression of dE2F2. Overexpression of dE2F2 alone decreased *RpL37a* promoter activity by about one-third, with a modest but reproducible ∼15–20% repression observed on *RpS29* and *mRpL22* promoters. These latter promoters were not sensitive to RBf1 or RBf2 overexpression alone. The *mRpL1* or *RpS14b* promoters were not repressed to any extent by any of the overexpressed proteins, and in fact transcription of these reporters was mildly stimulated. Thus, unlike the classical RB cell-cycle target *PCNA*, whose expression dynamically varies during cell growth, regulation of these non-canonical ribosomal protein gene promoters is more restrained. This behavior is consistent with the similarly modest but reproducible regulation of these genes under growth-limiting or stress conditions (Gasch *et al.* 2000; Causton *et al.* 2001; Gershman *et al.* 2007; Miller *et al.* 2011). As central mediators of global protein expression, small changes in ribosomal protein expression are predicted to have significant and pleiotropic effects (Steffen *et al.* 2012; Xue and Barna 2012; Woolford and Baserga 2013; Hasygar and Hietakangas 2014).

### RBf1 represses ribosomal protein gene expression *in vivo*

**Figure 4:**
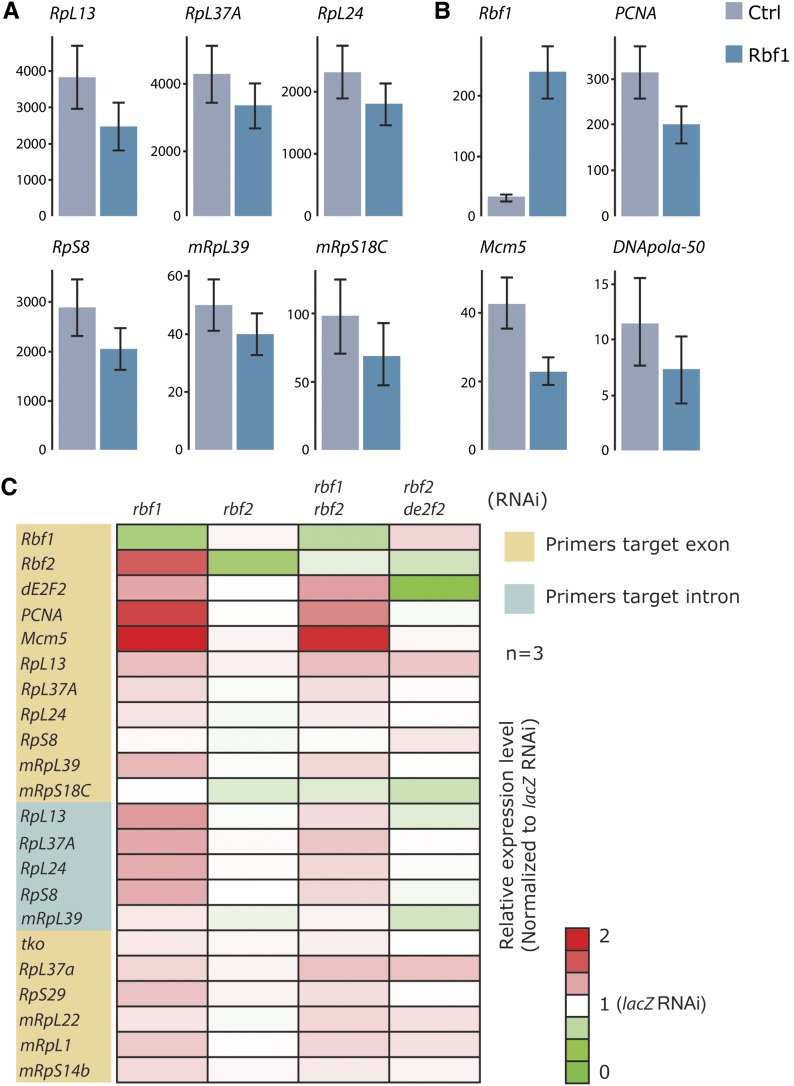
RBf1-mediated reduction of ribosomal protein gene expression in wing imaginal disc tissue. (A) Six ribosomal protein genes were significantly repressed in response to overexpression of RBf1 (p-value < 0.007, q-value < 0.05) (B) Cell-cycle genes were repressed by RBf1 overexpression. The y-axis indicates FPKM value (*i.e.*, fragments per kilobase of transcript per million), error bars indicate cross-replicate variability and measurement uncertainty (Trapnell *et al.* 2012). RBf1 was expressed in wing discs of third instar larvae under control of the *Pen* > Gal4 driver; three biological replicates were conducted and analyzed by RNA sequencing (RNA-seq), as described in the section *Materials and Methods*. (C) Ribosomal protein gene expression increased upon *RBf1* RNA interference (RNAi). S2 cells were treated with dsRNA for *lacZ* (negative control), *RBf1*, *RBf2*, *RBf1+RBf2*, and *RBf2+de2f2*. Gene expression for selected cell-cycle genes (*PCNA* and *Mcm5*) and ribosomal protein genes was detected by quantitative reverse transcription polymerase chain reaction. Consistent with the RNA-seq data for RBf1 overexpression, *RpL13*, *RpL37A*, *RpL24*, *RpS8*, *mRpL39*, and *mRpS18C* gene expressions increased upon *RBf1* or *RBf1+RBf2* RNAi. We also detected modest derepression of ribosomal protein genes *tko*, *RpL37a*, *RpS29*, *mRpL22*, *mRpL1*, and *mRpS14b*, even though luciferase reporters with these promoters were not repressed by ectopic RBf1 overexpression in transfected cells (Figure 3).

To further examine the significance of RBf1 association with ribosomal protein gene promoters, we performed RNA-seq of larval wing discs that were engineered to overexpress RBf1 (Elenbaas *et al.* 2014). Globally, a majority of the ribosomal protein genes showed modest reductions in expression, with only a few showing an increase (Figure S6). Six ribosomal protein genes were significantly repressed by RBf1 in this developmental context, showing decreases of 20–35% (Figure 4A), similar to the repression observed on cell cycle genes, including *PCNA*, *DNApolα-50*, and *Mcm5* (Figure 4B). Consistent with the RBf1 overexpression data, knocking-down *RBf1* alone, or *RBf1* with *RBf2* in cell culture significantly increased cell cycle genes expression, and widely induced ribosomal protein genes expression (Figure 4C). Knocking-down *RBf2* alone or together with *de2f2* did not have much impact on the ribosomal protein genes, although some of these gene promoters were significantly repressed by RBf2/dE2F2 *in vitro* (Figure 3). Interestingly, among this set of ribosomal protein genes, only *RpL13* was bound by RBf1 and RBf2 in embryos and larvae (Acharya *et al.* 2012; Korenjak *et al.* 2012; and this study). We speculate that some of these genes not found to bind the corepressor in the embryo may bind RBf1 specifically in the rapidly proliferating cells of the wing disc, or alternatively, these genes may harbor lower levels of RBf1 that were not called as peaks in our analysis. Indeed, a number of these promoters contain DNA motifs such as DREF and RAM that were also enriched under RBf1 peaks, and which may be diagnostic of RBf1 function (Acharya *et al.* 2012).

### Enrichment of BEAF-32 motifs in RBf-bound promoters

**Figure 5:**
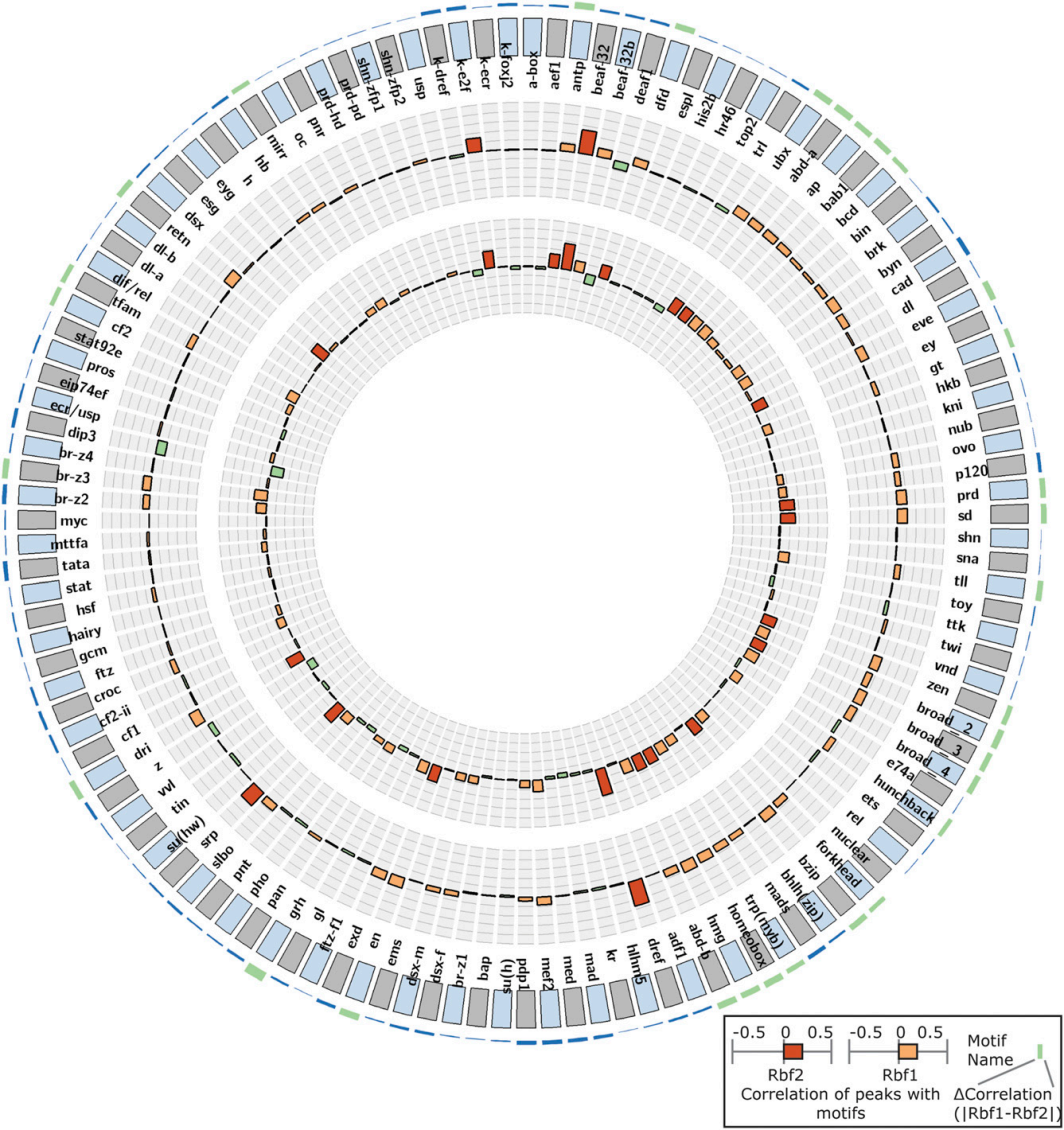
Enrichment of specific DNA binding protein motifs under peaks for RBf1 and RBf2 genome-wide. STAP results from 127 motifs were plotted in circular diagrams. The first histogram below the circumference shows Pearson correlation scores of individual motifs with RBf1 ChIP-exo peak intensity, and the inner histogram in the circle shows the RBf2 data. Strong enrichment for E2F, DREF, and BEAF-32 motifs is noted for both RBf1 and RBf2. The scale is from −0.5 to 0.5 with baseline of 0 in the middle, high scores (>0.19) are indicated in dark orange color (implying strong correlation), medium scores are in orange, and negative correlations are in green. The histogram outside the circumference shows the score differences between RBf1 and RBf2. The histogram is of light green color by default. Correlation-difference values lower than 0.04 are in blue color, implying those motifs are correlated with both RBf1 and RBf2 at similar level; correlation-difference values higher than 0.14 are in dark red color as seen in Figure S7, Figure S8, Figure S9, and Figure S10, implying those motifs correlate with RBf1-binding, but not RBf2-binding, and *vice versa*. The Pearson correlation scores were calculated on the whole fly genome. For data in the four functional classes (cell cycle, signaling, and ribosomal protein genes: cytoplasmic and mitochondrial), see Figure S7, Figure S8, Figure S9, and Figure S10. Raw numbers are available in File S4.

**Figure 6:**
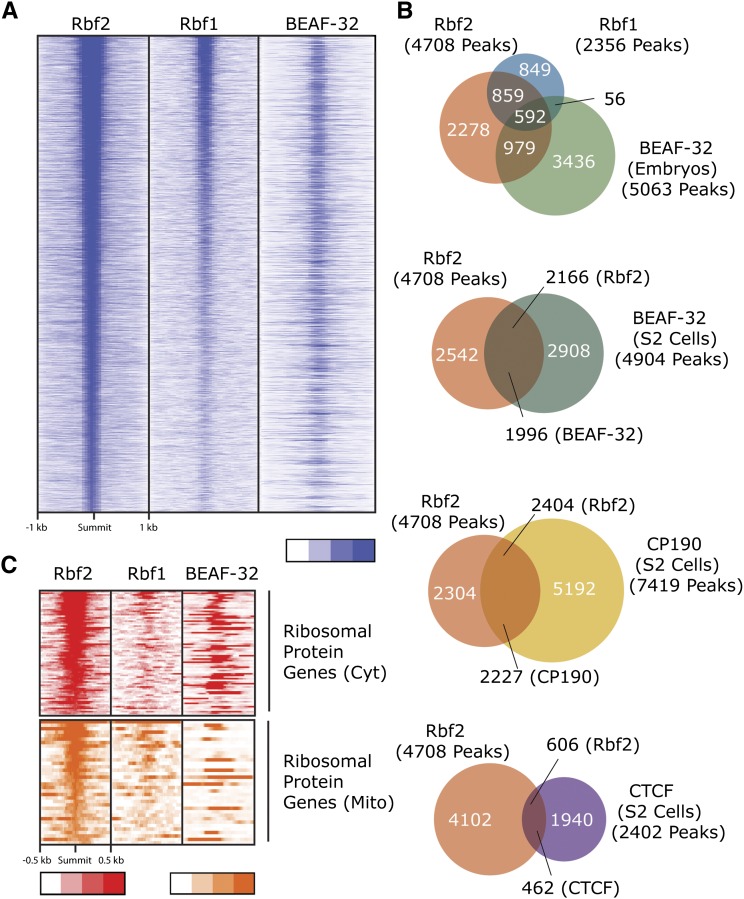
Correlation between RBf2, RBf1, and BEAF-32 ChIP signals (Yang *et al.* 2012). (A) Heat map centered on the RBf2 peak summits and sorted by RBf2 peak scores on all genomic regions bound by RBf2. (B) Comparison of RBf2, RBf1, BEAF-32 (embryos) (Yang *et al.* 2012), BEAF-32 (S2 cells), CP190 (S2 cells), and CTCF (S2 cells) (Schwartz *et al.* 2012) binding sites. The overlap between RBf2 and BEAF-32 (embryos) was statistically significant (log(p) = −5763). The statistics for significance of other overlap between RBf sites and insulator proteins (BEAF-32, CP190, CTCF, in S2 cells) is shown in File S5. (C) Correlations between positions of binding of RBf2, RBf1, and BEAF-32 are shown in ribosomal protein promoter regions, using heat maps centered on the RBf2 peak summits, and sorted by RBf2 peak scores.

Our analyses of the RBf and E2F genomic binding profiles revealed many RBf2 target genes that were not bound by E2F factors, and therefore we tested whether there was evidence for other transcription factors associated with RBf2 bound regions on target promoters. To identify relevant motifs, we used the STAP program, which correlates ChIP signal intensity with presence of overrepresented motifs for known transcription factors (He *et al.* 2009). Globally, a few motifs showed strong correlation with RBf1 and RBf2 peaks, including the E2F and DREF motifs that we previously demonstrated to be enriched at RBf1 binding sites (Acharya *et al.* 2012) (Figure 5). Viewed as separate classes, those genes annotated as “cell cycle related”, “signaling” and “cytoplasmic ribosomal protein” also showed a strong enrichment for the E2F motif (Figure S7, Figure S8, and Figure S9). Promoters from cell cycle and ribosomal protein genes also were enriched in a variety of other motifs, presumably related to their unique regulation (Figure S7, Figure S9, and Figure S10). However, genes representing conserved signaling pathways were not strongly enriched for additional motifs, likely because the divergent promoter sequences have very diverse regulatory properties (Figure S8). Unexpectedly, we found motifs for BEAF-32, an insulator binding protein, significantly correlated with both RBf1 and especially with RBf2 peaks (Figure 5). BEAF-32 binding sites measured in *Drosophila* 0-8-hr old embryos significantly overlap with RBf1 and RBf2 peaks (Figure 6, A and B), with co-occupancy found for one-third of the RBf2 and just over one-quarter of RBf1 sites (Figure 6B) (Yang *et al.* 2012). A similar overlapping was also observed for BEAF-32 binding sites in S2 cells (Figure 6B) (Schwartz *et al.* 2012). Other insulator proteins, such as CP190 also co-occupy RBf2 binding sites similar to BEAF-32, whereas the overlapping between RBf2 and CTCF was less significant (Figure 6B) (Schwartz *et al.* 2012). Focusing specifically on ribosomal protein gene promoters, BEAF-32 binding sites were significantly enriched, especially on RBf2-bound genes (Figure 6C).

### Some ribosomal protein gene promoters exhibit noncanonical RBf recruitment

**Figure 7:**
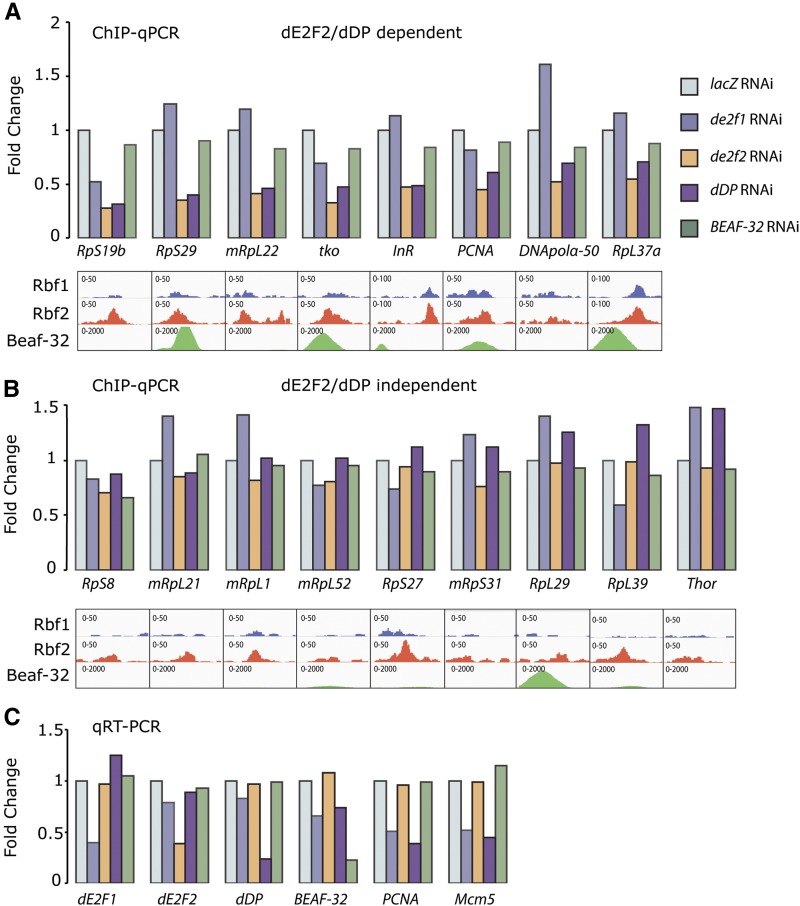
RNA interference (RNAi) depletion reveals E2F/DP-dependent and -independent RBf2 binding in cultured cells. (A) ChIP results for genes on which RBf2 binding to promoters was affected by *de2f2* or *dDP* knockdown. (B) Chromatin immunoprecipitation (ChIP) results for genes on which RBf2 showed little or no loss of binding by similar depletions. These promoters had weak or nonexistent RBf1 binding. ChIP recovery for factor depletion was normalized to levels obtained for *lacZ* control knockdown. (C) Knockdown efficiency of targeted mRNAs was ∼60–70%, as revealed by quantitative reverse-transcription polymerase chain reaction. Consistent with this depletion, the *de2f1* or *dDP* knockdown strongly affects the expression of *PCNA* and *Mcm5* cell-cycle genes.

Our discovery of genes uniquely bound by RBf2 but not RBf1, and the lack of E2F-like motifs within these promoter regions, prompted us to test whether RBf2 might be recruited to promoters by alternative factors. We tested whether RBf2 recruitment would therefore be dependent on dE2F/dDP proteins or BEAF-32 in cultured cells. We depleted *de2f1*, *de2f2*, *dDP*, or *BEAF-32* in *Drosophila* Kc cells with double-stranded RNA, followed by ChIP for RBf2. (Figure 7, A and B). The knockdown was sufficient to substantially deplete endogenous gene expression levels, leading to loss of expression of cell cycle genes *PCNA* and *Mcm5* in the cases of *de2f1* and *dDP* knockdown (Figure 7C). We examined promoters from cell cycle genes (*DNApolα-50*, *PCNA*), signaling pathway genes (*InR*, *Thor*), and ribosomal protein targets either bound by both RBf1 and RBf2 or RBf2 alone. Knockdown of *BEAF-32* had no effect on RBf2 recruitment on any promoter, even those with the greatest BEAF-32 binding signals (Figure 7, A and B). Thus, RBf2 and BEAF-32 may bind to these promoters independently. By contrast, knockdown of *de2f2* or *dDP* substantially reduced the RBf2 signal on the *RpS19b*, *RpS29*, *mRpL22*, *mRpS12/tko InR*, *PCNA*, *DNApolα-50*, and *RpL37a* promoters (Figure 7A), consistent with the previously described RBf2-dE2F2-dDP recruitment mechanism (Stevaux *et al.* 2002). Significantly, for the *Thor* gene and eight other ribosomal gene promoters tested, the *de2f2/dDP* knockdown showed little to no effect on RBf2 interaction (Figure 7B). It is interesting that most of those promoters were not bound by RBf1, and a previous study also suggested they were not bound by dDP (Ambrus *et al.* 2013). Interestingly, on a number of promoters, we observed a modest increase of RBf2 signal upon *de2f1* knockdown, possibly because of competition between dE2F2/RBf2 and dE2F1 on some RBf targets. We repeated this ChIP experiment in *Drosophila* S2 cells, and found that RBf2 binding on these RBf2-alone ribosomal protein gene promoters was also not affected by *de2f2/dDP* knockdown (data not shown). These results suggest that RBf2 interacts with some promoters via an E2F/DP-independent mechanism.

**Figure 8:**
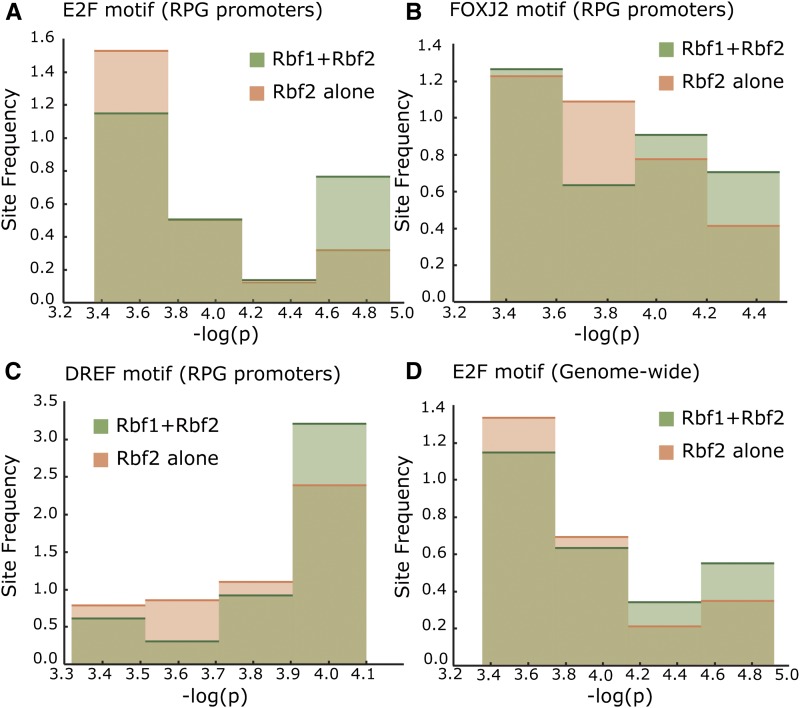
Distinct qualities of motifs associated with RBf1 + RBf2 bound promoters, *vs.* those bound solely by RBf2. (A) The E2F motif quality was greatest on ribosomal promoters bound by both RBf1 and RBf2; -log p values indicated on horizontal axis, and frequency of occurrence on vertical axis. (B, C) Previously identified RBf1-associated motifs DREF and FOXJ2 also show a tendency toward stronger sites in co-bound sequences. (D) The site strength of E2F motif was also found significantly shifted toward stronger sites in the RBf1 + RBf2 promoters, compared to the RBf2-alone promoters, when assessed genome-wide (*P* = 1.48 e-09). A total of 120 motifs were tested for differential representation in the two classes of RBf2 alone *vs.* RBf1+2; full results see File S9.

To determine whether ribosomal protein gene promoters bound preferentially by RBf2 may have different TFBS, we analyzed the occurrences and affinities of E2F-, DREF-, and FOXJ2-like motifs that previously had been shown to be enriched on RBf1 bound regions (Acharya *et al.* 2012). We found that promoters bound uniquely by RBf2 have lower binding scores for E2F, DREF, and FOXJ2 (Figure 8, A−C). Surveying the entire set of sites uniquely bound by RBf2 genome-wide, we found a similar lack of strong E2F sites (Figure 8D).

### Association with ribosomal protein gene promoters is a conserved character for the RB family

**Figure 9:**
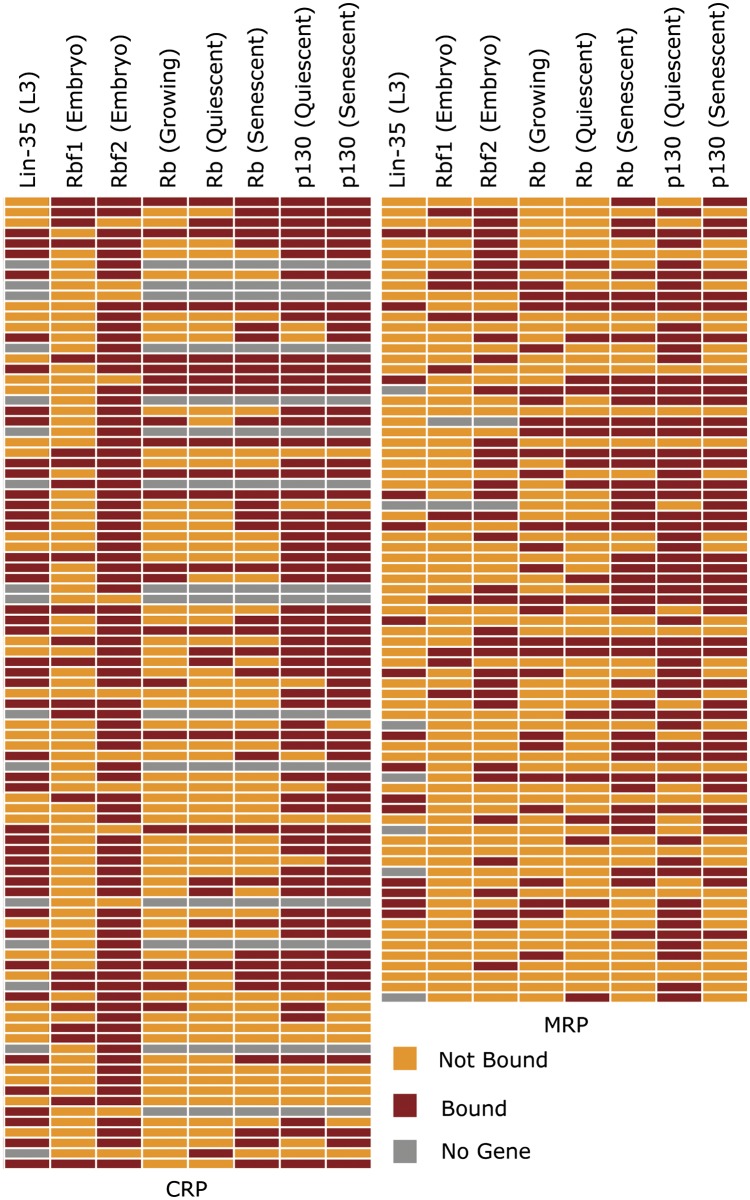
Retinoblastoma corepressor association with ribosomal protein genes is a conserved feature of RB proteins. Orthologous ribosomal protein genes were identified in *Caenorhabditis elegans*, *Drosophila*, and human, and association with retinoblastoma family proteins in promoter regions noted by colored lines. Association of retinoblastoma proteins is noted for a large fraction of cytoplasmic and mitochondrial promoters. Binding to each promoter from the *C. elegans* and human data set was analyzed by uploading peak calling files from Latorre *et al.* 2015, and Chicas *et al.* 2010, and manually annotating peaks within 500 bp of the transcription start site as shown in File S6.

To determine whether the widespread RBf association with ribosomal protein gene promoters represents conserved regulatory interactions, we surveyed human RB and p130 protein ChIP-seq data in fibroblasts (Chicas *et al.* 2010), and *C. elegans* RB homolog protein Lin-35 ChIP-seq in larvae (Latorre *et al.* 2015). We inspected all human and *C. elegans* orthologs of *Drosophila* ribosomal protein genes, observing that a majority of the ribosomal protein gene promoters were bound by RB, p130, or Lin-35 (Figure 9). The high proportion of ribosomal protein genes targeted by these corepressors suggests that there may be a conserved role for these RB family proteins in regulating protein synthesis and growth.

## Discussion

RB gene families have undergone diversification in multiple lineages (Cao *et al.* 2010; Gutzat *et al.* 2012). In metazoans, the RB family proteins of *Drosophila* and vertebrates have independently diversified; in the case of flies, RBf2 has substantial differences in the C-terminus, which is thought to be a key domain for regulation and binding specificity. In vertebrates, RB similarly exhibits substantial differences in the C-terminus compared with the more ancestral p107 and p130 paralogs (Classon and Dyson 2001). Thus, RBf2 and RB represent evolutionary innovations, which may direct the regulation of unique sets of genes or respond to different environmental and developmental signals.

The ChIP-Seq comparison of RBf1 and RBf2 binding profiles revealed several unexpected features, given previous findings that RBf2 cooccupies a number of promoters with RBf1. First, there were approximately 2000 genes targeted uniquely by RBf2. This pattern either represents the neofunctionalization of RBf2 with acquisition of novel gene targets, or alternatively, many of these genes may be bound by the RBf1 homolog in sister species, with a RBf2 acquiring some of these interactions through subfunctionalization of RBf1. Comparative functional studies will help to clarify this point. The unique binding of RBf2 to some promoters runs contrary to an earlier model that suggested that RBf2 would only interact with a subset of the genes bound by RBf1, because RBf2 was thought to bind preferentially to dE2F2, while RBf1 was more promiscuous. Our bioinformatic analysis indicates that there are indeed distinct patterns of motifs present on RBf2-only regions, including a depletion of strong E2F-like sites, suggesting that other transcription factors may direct RBf2 recruitment. In mammals, RB and p107 specificity is driven partially by differential contacts mediated by the C-terminal regulatory domains (Rubin *et al.* 2005; Julian *et al.* 2008; Cecchini and Dick 2011; Dick and Rubin 2013). Likewise, the unique C terminal domain of RBf2 may allow interactions with different types of regulators. Previous genetic experiments showed a genome-wide depletion of RBf1 binding in *dDP* mutant larvae, as well as loss of RBf2 from select genes. Our results are consistent with these findings, in that those specific genes tested for RBf2 association (such as *InR*) are E2F-dependent genes that are also bound by RBf1. Just as mammalian RB has diversified its interactions with the genome through association with non-E2F factors, *Drosophila* RBf2 may have alternative binding partners whose identities remain to be determined.

Despite the widespread binding of RBf2 in the *Drosophila* genome, genetic analysis of *RBf2* has shown that flies lacking this gene are viable, unlike the lethal phenotype of *RBf1* mutants. Why is the *RBf2* gene evolutionarily retained throughout the *Drosophila* lineage, despite the modest phenotype? The genes exhibit similar, although not identical expression patterns, suggesting that both proteins are likely to be present in many tissues. One clue comes from the adult pattern of *RBf2* expression, which is concentrated in the ovary (Stevaux *et al.* 2002; Keller *et al.* 2005). Although *RBf2* nulls were healthy and viable, these mutants lay eggs at a considerably higher rate than wild-type controls (Stevaux *et al.* 2005). Reproductive output is doubtlessly under strong selection, and *Drosophila* egg laying is in fact tightly coupled to nutritional signals. Excessive resource allocation represented by high rates of egg laying under laboratory conditions may be reproductively disadvantageous over the life span of the individual. Thus, the presence of RBf2 may modulate egg laying through fine-tuned transcriptional control of cellular signaling genes, as well as control of core biosynthetic components, such as the ribosomal protein gene family.

Our study suggests that RBf corepressors may be directly repressing transcription of ribosomal protein genes; interestingly, there is no precedent for direct negative regulation of this class of genes by transcriptional repressors. Previous studies have focused on the engagement of transcriptional activators at ribosomal protein gene promoters. In light of the central role that ribosome biogenesis plays in controlling global gene expression, it is rather surprising that this regulon would be controlled solely by positive inputs. Almost every regulated gene, from phages to bacteria to eukaryotic cells, features the combined action of both activators and repressors to achieve fine-tuned gene expression. The ribosomal protein genes represent a unique class that typically exhibits less variation in expression levels than developmentally-regulated genes, which may be completely silenced in many settings. Thus, typical transcriptional regulation of ribosomal protein genes may be rather subtle, but such modulation would nevertheless have pleiotropic consequences if not correctly executed. Global gene analyses typically focus on more dramatic fold changes than we observe here, thus this response may have been previously below the threshold considered to be significant (Dimova *et al.* 2003).

The selective regulation of ribosomal protein genes noted in our study, whereby only a subset of promoters was bound or regulated, is consistent with previous findings that the regulation of mRNA levels of some ribosomal protein genes is more dynamic than others, likely because other layers of regulation ensure stoichiometric production of ribosome components (Miller *et al.* 2011). The heterogeneous composition of activators at ribosomal promoters may contribute to this differential regulation; in mammals, the DRE motif for the DREF factor is found at many but not all ribosomal protein promoters, suggesting that common but not identical levels of regulation are probably at work (Yamashita *et al.* 2007). It is interesting that mammalian RB has been reported to directly regulate the activity of RNA polymerase I and III, providing a link for this cell-cycle regulatory protein to control the biosynthetic capacity of cells (Cavanaugh *et al.* 1995; Larminie *et al.* 1997; White 1997; Hirsch *et al.* 2000, 2004; Felton-Edkins *et al.* 2003; Gjidoda and Henry 2013). A regulatory connection with ribosomal protein genes would ensure that all facets of ribosome production would be influenced by RB signaling. Just as misregulation of c-Myc, which plays a positive role in ribosome synthesis, is linked to cancer, this model provides a new perspective to the impact of retinoblastoma proteins in cancer, where both disturbances to cell cycle control as well as accumulation of biomass through control of ribosome genes would play critical roles in tumorigenesis (White 2004, 2005).
